# Prevalence of Dementia in American Indian/ Alaska Native Medicare Beneficiaries

**DOI:** 10.1093/geroni/igab046.2772

**Published:** 2021-12-17

**Authors:** Heehyul Moon, Richard MacLehose, Joseph Kaholokula, Sunshine Rote, Soonhee Roh

**Affiliations:** 1 University of Louisville, university of Louisville, Kentucky, United States; 2 University of Minnesota, Minneapolis, Minnesota, United States; 3 John A. Burns School of Medicine, University of Hawaii at Manoa, Honolulu, Hawaii, United States; 4 University of Louisville, Louisville, Kentucky, United States; 5 University of South Dakota, Sioux Falls,, South Dakota, United States

## Abstract

Given the increase in life expectancy for American Indians and Alaska Natives (AIANs) due to many positive changes in social and environmental factors, the number of AIAN older adults with dementia is expected to grow from 23,850 in 2010 to over 100,000 by 2050. However, there have been few studies on the prevalence of dementia that have included AIANs . The purpose of the current study was to estimate the prevalence of dementia among AIANs over 65 years compared to non-Hispanic Whites (NHWs), Non-Hispanic Blacks (NHBs), Hispanics. The current study used survey data from Round 5 of the National Health and Aging Trends Study (NHATS, 2015) (N=7,449), a nationally representative study of Medicare beneficiaries 65 years and older. We estimated the age and gender-adjusted prevalence of dementia and 95% confidence intervals for each race/ethnicity. The majority of participants were between 65 and 74 years old. Slightly more than half of them were female. AIAN Medicare beneficiaries showed a significantly higher prevalence of dementia than NHWs after adjusting for age and gender (4% greater or higher prevalence). We also observed a significantly lower prevalence of dementia in AIAN Medicare beneficiaries than NHBs, Hispanics. While previous research has reported that AIANs shave a similar or lower prevalence of dementia than NHWs, our findings indicate significant dementia disparities in AIAN Medicare beneficiaries. Future research should focus on dementia prevalence and risk factors within/between AIAN tribes.

